# Satellite License Plate: passive and compact optical spectrally-based identification method for satellites

**DOI:** 10.1038/s44172-024-00188-2

**Published:** 2024-03-20

**Authors:** David L. Bakker, Gustavo Castro do Amaral, Eugenio Di Iorio, Linda W. Feenstra, Ivan Ferrario, Breno Perlingeiro, Fabrizio Silvestri

**Affiliations:** High Tech Industry Unit, TNO, Stieltjesweg 1, Delft, 2628 CK The Netherlands

**Keywords:** Aerospace engineering, Astronomical optics, Fibre optics and optical communications

## Abstract

Satellite identification and tracking is fundamental for decision making in space traffic management. Cooperative optical identification methods enlarge the toolbox of identification techniques which currently count on radar and passive optical observations. Here we present a cooperative method to identify satellites from the ground by means of laser techniques: the Satellite License Plate (SLP). SLP employs unique spectrally-encoded retroreflecting tags mounted on the satellite. The interrogation of the tags could be performed with laser enabled optical ground stations. The benefit of the concept is that the tag concept is fully passive, minimally invasive, and scalable from tens to hundreds of unique combinations, allowing to map a large number of satellites on a single orbit. The SLP method is here described by means of end-to-end theoretical analyses on the final performance of identification and experimental results gathered during km-range ground-to-ground free space tests.

## Abstract (web summary)

A research team from the High-Tech Industry Unit at TNO reports a method to identify satellites using retroreflecting tags with spectral signatures mounted on these satellites. The approach reduces the complexity of the observation system and does not require any power sources on board.

## Introduction

The recent growth in launching commercial and institutional satellites has exposed the world to the issue of space traffic management. According to recent statistics, the number of satellite launches is increasing following an exponential trend in the last years, from about 100 launches per year in 2010, to more than ten times more in 2020^1^. With the Earth’s orbits getting crowded, the number of potential collisions between space objects is increasing. This issue has recently been calling for policies, best practices and technological solutions in terms of Space Traffic Management. Amongst these, the identification of space objects and debris is a first fundamental step. The capability of identifying a satellite could potentially help institutions or commercial service providers to recognise threatening collision situations and resolve them. In addition, in the case of satellite swarm launches, the identification of single swarm element could help in the characterization of the behavior of the satellite, before all satellites are fully operational. Nowadays, in fact it is not possible to identify a single satellite in a swarm until a radio contact is established.

The gravity of the issue increases when dealing with debris originated from fragmentation of space objects: out of 26,000 objects monitored, about 21,000 are unidentified debris objects of fragments^2^. Being able to identify debris objects consistently could potentially provide inputs for trajectory predictors.

Current identification technologies are based on RADAR observations^3^ and optical observations^4^. Still, a number of flying objects on LEO cannot be identified and, with the growing number of LEO launches, this number is expected to increase in absolute terms^5^. This outlook calls for additional research on spacecraft identification techniques from ground. The current identification process is based on a typical satellite signature: the trajectory and/or the time evolution of object’s light curve. These attempts of identification based on classification methods are attractive because they rely on already established infrastructures, and because, in principle, they could enable cooperative and non-cooperative identification.

Alternatively, a series of different methodologies have been proposed for cooperative identification, based on a more direct approach, which do not resort on the inversion and classification of indirect measurements. Cooperative identification methods include all strategies which foresee the presence of hardware on the satellite to be identified, which enables the identification process. Cooperative method in turn can be categorized in active and passive. Active techniques involve the use of a payload latched onto the spacecraft power bus. This is the case of the extremely low-resource optical identifier (ELROI), where a “license plate” consisting of an array of laser diodes is mounted on the satellite^6^. Each satellite equipped with the ELROI could be identified once the optical code sent to Earth is properly received and decoded. Although the ELROI solution could in principle produce a large number of unique identifiers, therefore covering a large number of satellites, it suffers from two drawbacks. It requires a continuous power supply, which is a limited resource for small satellites. And, to allow the identification also in the case the satellite ceases to work, a power supply independent from the main operational power supply needs to be added to the satellite payload.

Passive techniques are more attractive, because they do not require power supply from the satellite, and they allow the identification also when the satellite is not operational anymore. Passive cooperative techniques are mostly based on the presence of unique retroreflecting tags on the satellite, which can be identified from ground by means of properly designed optical ground stations. Based on the different physical properties of light, various methods have been devised. A basic identification scheme is based on the number of retroreflectors present on the satellite^7^. The level of the retroreflected signal is proportional to the number of retroreflectors, and therefore a unique identifier can be defined with the number of retroreflectors present on the flying body. Hence, to cover a large number of satellites the needed surface can grow quite rapidly on the satellite. The polarization properties of the retroreflectors can also be tuned to define unique identifiers^8^. In this way, with a limited number of retroreflectors on the satellite, a larger number of unique identifiers can be defined, exploiting the orthogonality of different polarization stages.

As a way to broaden the landscape of cooperative solutions, we have recently introduced a technique, based on the orthogonality of light response based on spectral properties of the retroreflecting tag^9^. Exploiting the spectral properties of the light to define unique satellite identifiers has several advantages: the tag can be composed of simple components like corner cube retroreflectors (CCRs) and band-pass filters (BPFs). Compared to the polarization-based identification^8^, the complexity of the interrogating ground station is simplified. In fact, it only requires the multiplexing of several, spectrally separated, laser beams at the transmitter side, and demultiplexing in the receiving part. This method does not require time-division-based interrogation, and the read-out of the spectrally separated retroreflectors can be done in parallel. This allows to employ the full visibility time interval for the interrogation of all spectral channels, increasing the size of accessible data for the identification.

This paper is devoted to a detailed introduction and explanation of the Satellite License Plate (SLP) concept. The identification process is described together with the hardware involved. Moreover, end-to-end analysis results on the feasibility of the method in a LEO satellite scenario are also reported. We built upon the concept introduced in^9^ to experimentally demonstrate the SLP method in a ground-to-ground free-space test with increased representativeness with respect to the ground-to-satellite case.

## Results

### Satellite License Plate concept

**Fig. 1:**
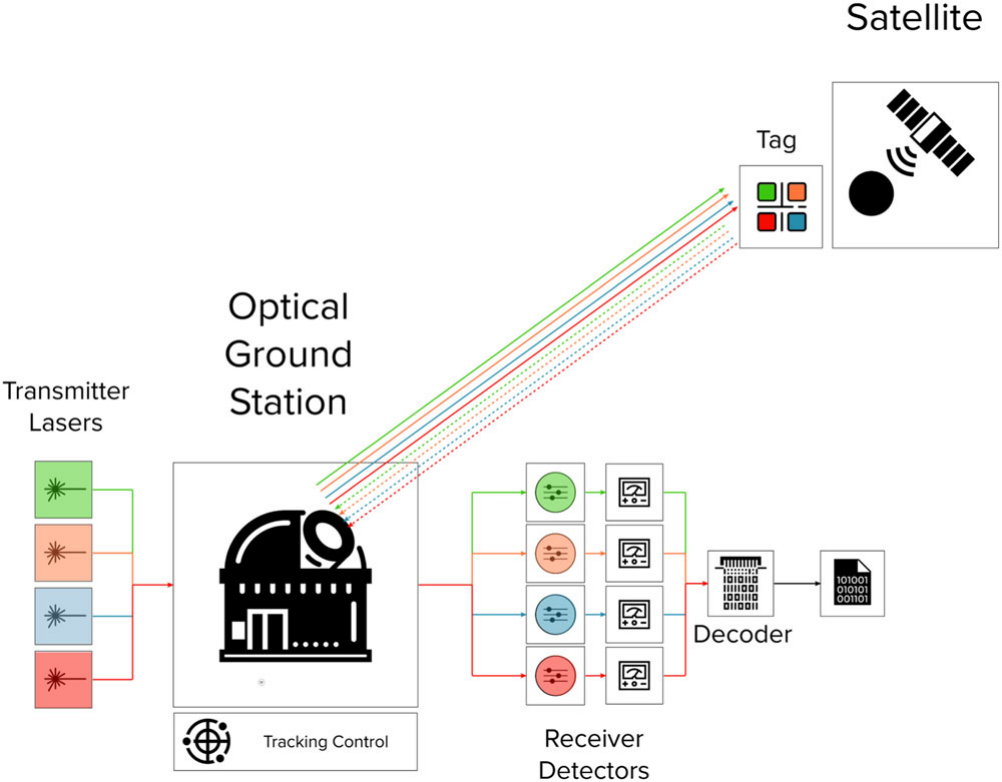
Satellite License Plate cooperative identification method concept. A satellite with a spectrally-encoded Tag on-board is interrogated by a multi-wavelength laser beam from the Optical Ground Station. The Received signal is spectrally separated and decoded to retrieve the unique spectral signature of the Tag.

**Fig. 2:**
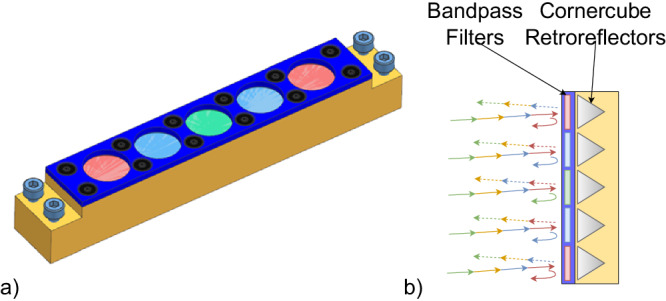
SLP tag assembly. 3D render of a possible implementation, dimensions 14 mm  × 14 mm  × 93 mm (**a**), cross-section of the tag assembly consisting of bandpass filters and cornercube retroreflectors (**b**). The working principle of the tag is sketched using solid lines to represent the multiwavelength interrogation beam, dashed lines for the specular reflection and curved-solid lines to represent the retroreflected spectral beam.

The SLP concept is pictorially described in Fig. 1. In order to establish a cooperative identification, a retroreflecting tag with a spectrally encoded identifier should be mounted on the satellite, as shown in Fig. 2a, b. To enhance the visibility chances, multiple instances of the same identifier should be mounted on the different faces of the satellite. In the case of low-Earth orbit (LEO) satellite targets, the suggested concept can work with the 1/2″ or 1″ version Corner Cube Retroreflector (CCR) ranging between 10 mm to 30 mm in diameters, limiting the volume of the tag to tens of cm^3^ (for the assembly shown in Fig. 2a, b the expected volume is 18 cm^3^). Most of the system complexity of the identification method resides in the optical ground station (OGS). In order to establish a line of sight with the satellite, a narrow field of view (FOV) telescope capable of tracking objects in LEO is used and the initial orbital parameters are obtained from the ephemeris table provided by the satellite operator. Passive optical tracking on initial satellite passes is used to correct the residual uncertainty on the orbital parameters and, from the further passes, it is possible to start with the interrogation of the license plate^10^. The orbit prediction is used to feed the OGS control system, to provide the tracking trajectory during the interrogation of the SUT. The transmitter of the OGS contains a series of laser emitters, whose characteristic wavelengths are spaced at least tens of nanometers, optics for collimation and multiplexing and a beam launcher. The latter is typically an additional afocal telescope, to be connected to the main larger aperture telescope of the OGS. The bi-static assembly of the OGS allows for proper separation of the laser light between the transmitter and receiver path, while still sharing the same mechanics and control features, allowing for good co-alignment between the two paths. The light beams returning from the SUT are collected by the telescope aperture, steered to the receiver and then spectrally demultiplexed before reaching the detection and decoding unit. This contains spectrally separated photodetectors (e.g., avalanche photodiodes), analog-to-digital converters and a digital decoding unit. This unit is responsible for converting the electro-optical signals into the most likely identifier, which is assigned to the SUT.

The identification scheme is based on arrays of spectrally selective retroreflecting tags. These consist of groups of BPFs and CCRs stacked together. The working principle of the tag, and a possible realization of it, is sketched in Fig. 2. When illuminated by a multispectral interrogating beam, each BPF will transmit the portion of light contained in its characteristic bandwidth. The rest of the light is either reflected, or absorbed, in case of absorptive coating. The transmitted light will be retroreflected by the CCR and transmitted back to the OGS through the BPF. In this way, the amount of light returned to the OGS, as function of the spectral bandwidth, is linearly dependent to the number of CCR covered by the respective BPF. Although not shown in Fig. 2a, b, a reference CCR without any BPF, should be inserted in the tag, to provide a radiometric reference independent from the wavelength of excitation. The presented tag design has the main advantage of no cross-section reduction when the interrogation beam is incident on-axis. However, for off-axis interrogation, a reduction of cross-section needs to be taken into account, which is translated as the angular response of the tag—refer to the section “Ground-to-ground free space tests: experimental results”—for the experimentally determined angular response. The signal collected by the OGS *S*_*i*_, per wavelength is described by

1 $${S}_{i}\propto {T}_{i}^{2}{R}_{CCR}{N}_{i}+{C}_{i}^{2}{R}_{CCR}(N-{N}_{i}-1)+{R}_{CCR}\quad i=1,\ldots ,{L}_{\lambda }$$

with the constraint that

2 $$\mathop{\sum }\limits_{i=1}^{{L}_{\lambda }}{N}_{i}=N-1$$

where *L*_*λ*_ is the total number of spectral channels employed in the tag, *N* the total number of CCRs in the tag, *T*_*i*_ the in-band transmission of the BPF in the *i*-th channel, *C*_*i*_ the out-of-band (cross-coupling) transmission of the BPF in the *i*-th channel and *R*_*CCR*_ the effective reflectance of the CCRs. The spectral signature which can be received by the OGS is composed of a vector of ordered ratios, like

3 $${{{{{{{\boldsymbol{\rho }}}}}}}}=[{\rho }_{1| 2},\,{\rho }_{1| 3},\,\ldots {\rho }_{i| j},\,\ldots {\rho }_{({L}_{\lambda }-1)| {L}_{\lambda }}],\quad {\rho }_{i| j}=\frac{{S}_{i}-{S}_{j}}{{S}_{i}+{S}_{j}}$$

with the indices *i* = 1, …, *L*_*λ*_ − 1, *j* = (*i* + 1), …, *L*_*λ*_. Each received signature will be compared with the dictionary of all the possible allowable tag words determined by the used SLP configuration, which is given by the values (*N*, *L*_*λ*_). The amount of unique identifiers in a dictionary can be found employing combinatorial calculus^11^

4 $$Q=\left(\begin{array}{c}N+{L}_{\lambda }-2\\ N-1\end{array}\right).$$

For a case in which a total of *N* = 5 CCRs, of which 1 reference, and *L*_*λ*_ = 4 spectral channels are used, the total number of unique IDs in a dictionary is equal to 35. This easily scales up to more than a hundred unique identifiers, if for example arrays with *N* = 8 CCRs and *L*_*λ*_ = 4 spectral channels are used. However, even if a limited complexity tag dictionary with reduced number of CCRs and spectral channels is used, the amount of satellites covered by the identification is potentially larger. If the cooperative identification is complemented with information on the satellite orbit, e.g., satellite laser ranging (SLR) or satellite period extraction from observations, then the same tag could be at two different satellites, provided that their orbit can be discriminated. This suggests that the use of SLP could be scaled up, even if a limited dictionary of tens of unique tags is used.

In normal operation, the spectral signature of Equation (3) will be corrupted by electronic and background noise, and general fluctuations, instabilities of the system. A decision scheme based on Maximum Likelihood principle could be employed to assign an identifier to a SUT based on the received spectral signature.

### LEO satellite identification: end-to-end performance analysis

**Fig. 3:**
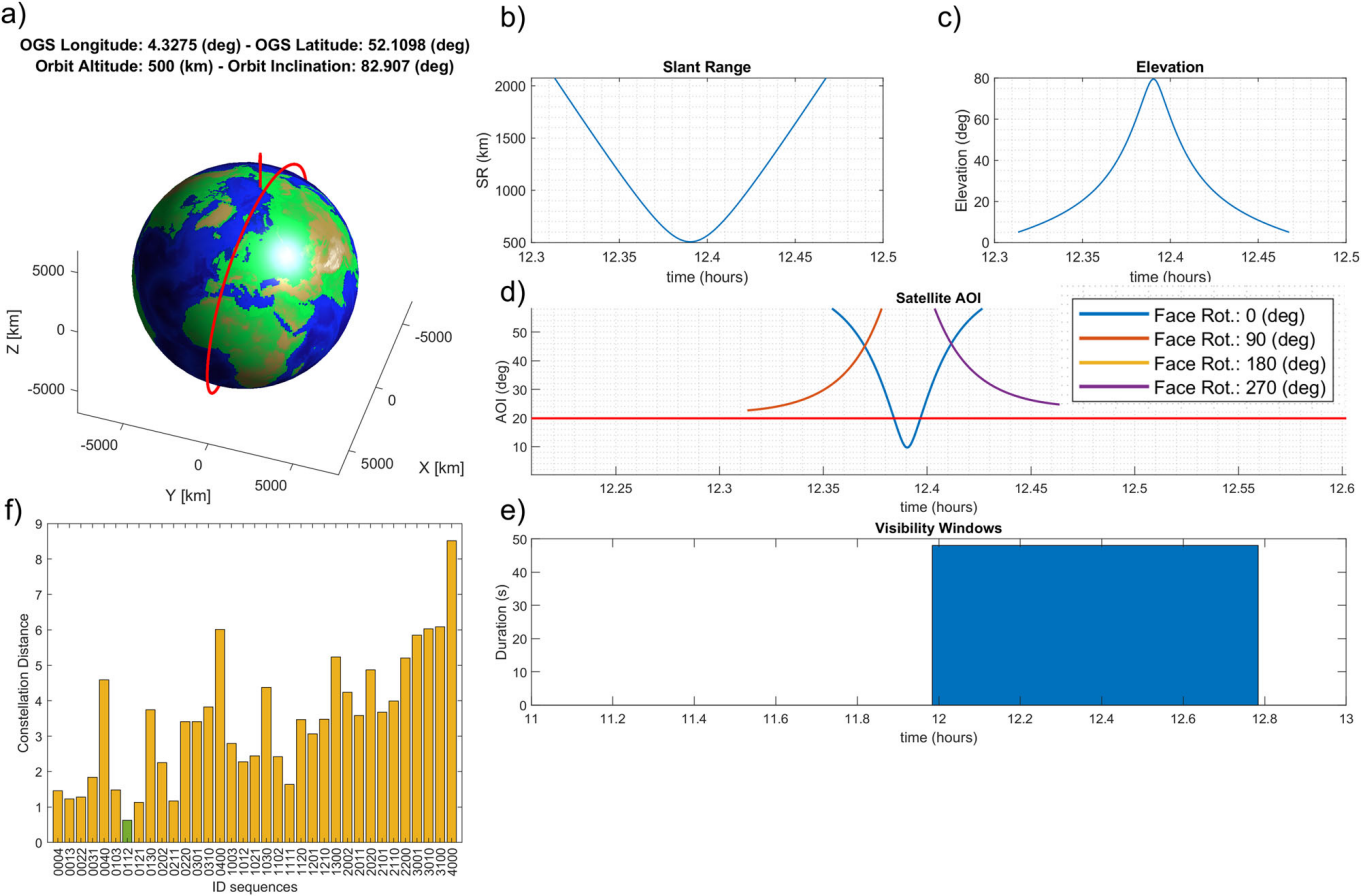
Numerical results of the end-to-end system modeling. 3D representation of the satellite trajectory simulated (**a**), derived slant range and satellite elevation (**b**, **c**), profile of angle of incidence (AOI) on the satellite tag (**d**), duration of the visibility time window (**e**). Maximum Likelihood decoding results expressed as norm-2 distance, the received spectral signature with respect nominal signatures, for the identifier $\left\{0112\right\}$ (**f**). The green color indicates the nominal tag code whose norm-2 distance from the received signal is minimum with respect all other distances. The reference time for the simulation is 14-06-2022 at 00.00. OGS, Optical Ground Station.

Numerical simulations have been performed to assess the viability of the concept, characterizing the end-to-end performance of the system. The numerical simulation is constructed in two phases: at first, the trajectory of a SUT is propagated in time, and the time interval of satellite visibility is calculated. This is determined by the instantaneous viewing geometry, geometric characteristics of the tag (angle of acceptance), and operational constraints of the OGS (elevation limitations). The result from this first step is fed into a time-based simulation of the signal propagation. In the simulation, the amplitude-modulated optical signal is propagated from the OGS towards the tag and back, and then all the way down to the opto-electrical conversion and decoding. A detailed description of the models involved in this simulation is reported in “Methods” section. The simulation considers a generic LEO satellite which orbits around Earth at an altitude of 500 km. The other simulation parameters, used to characterize the different components of the OGS and of the tag, are reported in SI-Table 1, available in the Supplementary Note 1. An example of the results of the numerical calculations is presented in Fig. 3a–f. In the plot each possible identifier is labeled with a digit string $\{{d}_{{\lambda }_{1}}{d}_{{\lambda }_{2}}{d}_{{\lambda }_{3}}{d}_{{\lambda }_{4}}\}$, where each digit represents the number of BPFs present on the tag for each spectral channel. The identification scheme considered in the numerical simulation is characterized by *N* = 4 + 1 retroreflectors, and *L*_*λ*_ = 4 spectral channels. Each of the 35 tags available within this scheme has been simulated. For each tag an attempt of identification is made based on the maximum likelihood approach, calculating the norm-2 distance between the received spectral signature and the nominal identifier signatures. The case presented in Fig. 3f shows a successful decoding of the tag. The simulated received spectral signature is in fact the one at minimum distance from the nominal signature of the tag simulated, {0112}. Over all possible tags, the numerical simulation reported a success rate of 97%, where only one out of a total of 35 tags was incorrectly decoded.

In the simulation the BPFs were modeled with an ideal response. However, their response is also dependent on the angle of incidence^12^. The angular response of the coating causes a blue-shift of the characteristic BPF response. The transmission of the BPF for a given channel, with central wavelength *λ*_*i*_, as function of the angle of incidence can be described as

5 $${T}_{i}(\lambda ;{\theta }_{inc})=\exp \left\{-2\frac{{\left[(\lambda -{\lambda }_{i}){\left(\frac{\sin ({\theta }_{inc})}{{n}_{eff}}\right)}^{2}\right]}^{2}}{{{{{{{{{\rm{BW}}}}}}}}}^{2}}\right\}$$

where BW is the 1/*e*^2^ half-bandwidth of the filter, *θ*_*inc*_ the angle of incidence, and *n*_*eff*_ the effective refractive index of the BPF coating stack, which models the angular dependency of the filter. The formulation here proposed, and described in more detail in Supplementary Note 2, can be used to properly define the spectral spacing between channels. The spectral channels should be spaced enough to avoid cross-coupling between each other, even at large angles of incidence. For example, with a filter with *n*_*eff*_ = 2.5, and *BW* = 5 nm, at 30^∘^ of angular incidence a spacing of 15 nm between the channels is enough to ensure that the maximum cross-coupling transmission is below −72 dB. The spectral channel spacing in the simulation has been set to this value, to ensure that no cross-coupling effect needs to be included in the model. As a practical consideration, it must be noted that introducing larger channel spacings will further reduce the cross-coupling, however, a too large spacing should be avoided. In fact, the overall bandwdith of the system, including all laser channels employed, should be kept limited within tens to hundreds of nanometers. Otherwise, the complexity of the optical system of the OGS transmitter could increase, to handle different spectral beams within the same system.

### Ground-to-ground free space tests: test description

Free space experimental tests have been conducted as proof of principle of the satellite identification proposed with the SLP method. The goal of the tests is to show the feasibility of unique identification and to characterize the identification process under representative conditions. Moreover, to show the maturity of the technology employed, it needs to be highlighted that this result is achieved with only off-the-shelf components. Recently^9^, a preliminary setup for ground-to-ground tests to demonstrate the concept of satellite optical spectrally-based identification was presented. This initial setup suffered from several limitations, of which the main ones were: the short path length available for the field-test (150 meters); and a single wavelength transceiver design (requiring time-multiplexed tag evaluation). These two issues, which were responsible for inducing artefacts in the initial measurements, have been solved by upgrading the transceiver optical head, allowing for a longer path length field-test (2500 meters), upgrading the optoelectronics design and hardware, enabling a wavelength-multiplexing scheme on the transceiver, as described in the section “Ground-to-ground free space test: system description”, and, thus, parallel two-channel interrogation, as well as optimizing the waveform shape and the data processing steps (e.g., the confusion matrix formalism). The remote identification test has been conducted between two locations in The Hague (NL), with a free space propagation path of 2.43 km (one-way). The SLP dictionary implemented was based on *L*_*λ*_ = 2 spectral channel, centered at 1540 nm and 1560 nm, and an array of *N* = 4 + 1 CCRs. In agreement with Equation (4), the number of unique identifiers for the tested identification is equal to 5.

**Fig. 4:**
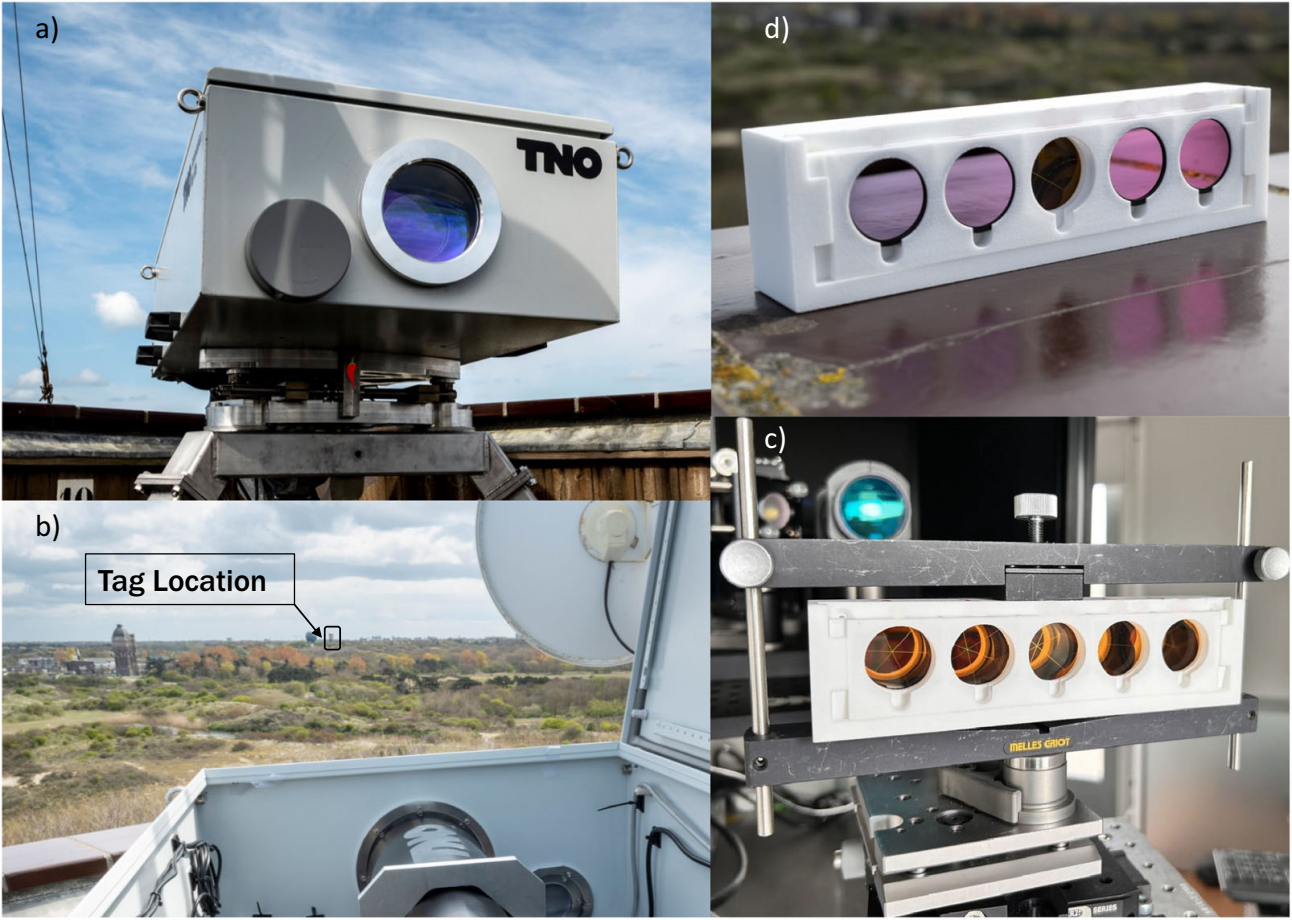
SLP field test setup. Mono-static OGS transceiver installed at location (**a**), free-space line of sight as seen from the Optical Ground Station transceiver location (**b**), SLP tag with unfiltered array of Corner Cube Retroreflectors installed in the support holder (**c**), example of a SLP tag with given combination of Band-Pass Filters (**d**). *Pictures courtesy of Eric de Vries*.

As shown in Fig. 4a, b, the OGS transceiver is implemented with a mono-static optical bench, where the transmitted and received beams pass through the same aperture. This bench was installed on top of a tower building at 37.5 m elevation while the CCR tag was installed at TNO The Hague premises, at a height of 40 m. The tag holder was designed for a quick replacement of BPFs, to allow a quick swap amongst different identifiers, as shown in Fig. 4c, d. The detailed description of the OGS transceiver is found in the “Methods” section.

The test was conducted in two phases: in the first one, all the dictionary identifiers were interrogated in sequence. This sequence included also a tag without BPFs. This configuration provided a reference measurement for calibration purposes. For each test configuration, the received signals were processed, following the procedure reported in the section “Ground-to-ground tests: data analysis”. Finally the processed results were compared with the expected nominal spectral signatures, to assess the quality of the identification. During this test the tag was kept fixed in position with an orientation perpendicular to the incoming beams.

The second phase of the test involved the characterization of the angular response of the tag. For this test a single configuration was tested. During the interrogation of the tag, the tag was rotated stepwise on the azimuthal plane and the return signals were recorded for each angular position (detailed procedure in the section “Ground-to-ground tests: procedures”).

### Ground-to-ground free space tests: experimental results

The BPFs chosen for the test were characterized by negligible cross-coupling (< −32 dB) and by similar high transmission (>95%). For the tested case, Equation (1) can be simplified assuming *C*_*i*_ ≈ 0 for *i* = 1, 2 and *T*_*i*_ ≈ *T*_*j*_ = *T*. By combining Equations (3) and (2) for the case *L*_*λ*_ = 2 and *N* = 5, it can be seen that the spectral signature for the experimental case is a single scalar value, which scales linearly with the number of BPFs present in the tag under test.

6 $${{{{{{{\boldsymbol{\rho }}}}}}}}={\rho }_{1| 2}=\frac{{T}^{2}(2{N}_{i}-N+1)}{{T}^{2}(N-1)+2}\approx \frac{2{N}_{1}}{N-1+2}=\frac{1}{3}{N}_{1}-\frac{2}{3}$$

The test plan involved multiple of the so-called “dictionary run”, which is characterized by five measurement step: one measurement step consists of a pre-defined tag interrogation period (200 seconds for round A and 600 seconds for round B), where the probe beam is launched towards the target from the OGS and several retroreflected waveforms are recorded for statistical analysis; different rounds correspond to a new tag configuration, where, without loss of generality, the following sequence was established $\left[\{00\},\{40\},\{31\},\{22\},\{13\},\{04\}\right]$, where the first index corresponds to the number of BPFs centered at *λ*_1_ = 1540 nm, and the second to those centered at *λ*_2_ = 1560 nm. The sequence of experimental results could be then compared to the linear curve described by Equation (6).

**Fig. 5:**
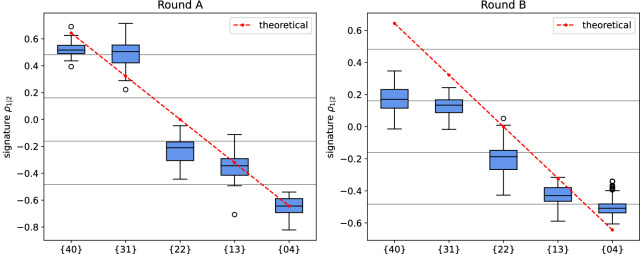
SLP dictionary test results. Independent measurements rounds A and B are reported. The boxplots are characterized by box size ranging from the first to third quartile value, with whiskers range equal to 1.5 times the quartile range. They are calculated respectively on 20 (Round A) and 100 (Round B) signal traces for each configuration point. The red dashed curve is the linear reference behavior as described by Equation (6).

**Fig. 6:**
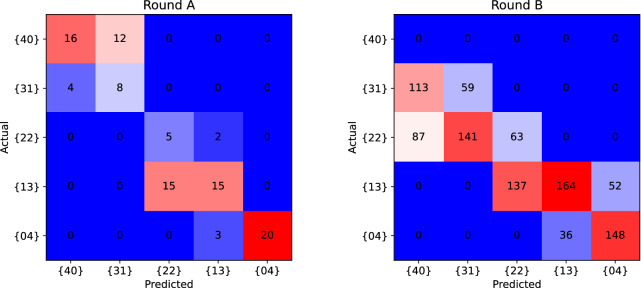
Confusion matrices for measurement rounds A and B. The x-axis shows the predicted identifier tag and the y-axis the actual identifier tag.

The results of the dictionary tests are reported in Fig. 5a, b, where the reference measurement (the {00} configuration) of each round is not depicted. The particular sequence chosen for the rounds is useful since it translates into a monotonically decreasing linear profile when mapped into Equation (6). The statistics extracted from the recorded waveforms are summarized with a standard boxplot, depicting the median as a black line. In an ideal scenario, the boxplot corresponding to a given round should fall entirely within the Voronoi region assigned to its respective tag configuration; the former is depicted in Fig. 5a, b as thin horizontal gray lines. Although the results do not follow the modeled scenario, a clear distinction between the rounds can be appreciated in the results, which follow reasonably well the trend of Equation (6). On the other hand, the signature variation extends over an interval larger than the Voronoi region assigned to the nominal tag configuration for unambiguous identification^13^. In order to deepen the analysis of the measurement campaign the formalism of Confusion Matrices is employed. These matrices are reported in Fig. 6a, b and they show how the predicted tag identifiers distribute against the actual identifiers. A robust identification prediction should display a confusion matrix where high-frequency values are concentrated on the diagonal. From the confusion matrices in Fig. 6a, b for dictionary run A, one can extract performance figures of merit, where the Matthew’s Correlation Coefficient (MCC) is the most relevant, since it allows evaluating a predictor’s performance^14^. The analysis produces averaged MCCs of 0.55 and 0.28 for rounds A and B, respectively—refer to SI-Table 2. According to the MCC interpretation, the test runs exhibit strong positive correlations for round A and weak positive correlations for round B. While having distinct MCC scores for the two rounds, it is visible that both dictionary signatures contain information about the tag, e.g., the monotonicity of the ordered profiles, as shown in Fig. 5a, b.

**Fig. 7:**
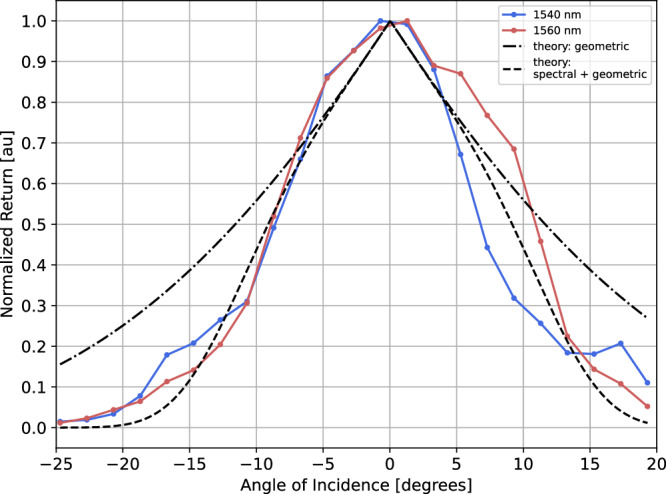
SLP tag angular response test results. Normalized profiles of the return signal as function of the angle of incidence of the interrogation beams on the tag. For reference four theoretical profiles are also shown, two for each spectral line. The one with ‘-.’ lines are the ones describing the angular response based only on geometric arguments, as described in ref. ^15^. The curves with ‘--’ lines are obtained considering the geometric loss and in addition the BPF angular response, as described in Equation (5).

The angular response of the tag was tested on the tag with configuration {22}. The signal return profiles, normalized with respect to their maximum, are reported in Fig. 7. In this figure two type of theoretical profiles are also reported for comparison. One profile is calculated following the mathematical formulation presented in ref. ^15^, which models the optical cross-section reduction of a CCR as function of the angle, based on geometrical arguments. A second theoretical profile is constructed including the geometric loss already mentioned, and the additional angular-dependent reduction due to the BPF angular response, as modeled in Equation (5). For the latter, the effective refractive index, *n*_*eff*_ = 2.5, and the 1/*e*^2^ bandwidth, *BW* = 10.7 nm, were obtained with a model fitting procedure based on data-sheet and measured data. From the comparison it is clear that the geometric loss alone cannot fully model the angular response of the measured tag. On the opposite the addition of the BPF response in the modeling curve allows to reach results close to the measured data, confirming the suggested behavioral model.

## Discussion

The experimental results obtained in the free-space ground-to-ground test demonstrate the working principle of the proposed Satellite License Plate identification method. Especially they show that unique identification of remote target is feasible, with off-the-shelf technology. The large statistical spread exhibited by the dictionary results can be ascribed to atmospheric channel variations and system drifts. The first type of variation is unavoidable, although it can be expected that in a final implementation, long averaging over multiple satellite passes, could help to reduce the variation. In terms of system drifts, the absence of an in-line calibration path in the OGS transceiver prevented a full compensation of fluctuations of the laser power levels, detector drifts and pointing instabilities. By just tapping a portion of the interrogation light before launching it in free space an in-line calibration path can be implemented, which could give real-time correction for the spectral signature.

An additional potential solution to increase the identification yield is the reduction of the dictionary granularity. The minimum variation in number of BPFs for spectral channel is equal to one. If this is increased, the unambiguous Voronoi regions of the signatures for each tag are extended, accommodating for larger measurement spread. However, this results in a reduction of the total number of combinations available. For example, considering a minimum spacing of two BPFs per spectral channel, Equation (4) should be modified as

7 $$Q=\left(\begin{array}{c}\lceil {\log }_{2}(N)+{L}_{\lambda }-2\rceil \\ \lceil {\log }_{2}(N)-1\rceil \end{array}\right),$$

where “⌈ ⋅ ⌉” represents the ceiling operator. For example, for *N* = 5 and *L*_*λ*_ = 4, the number of total combinations will reduce from the initial 35 to 10. Of course, the same improvement in noise-robustness could be obtained by increasing the size of the CCR apertures. However, this solution comes at the expense of the tag volume, which could be an issue for small satellites. In particular for the results of the ground-to-ground free space tests reported previously, relevant performance improvements are achieved when tag configurations {31} and {13} are removed from the analysis, as seen in the MCC evaluation predictor—in particular for round A—depicted in SI-Table 2 of the Supplementary Note 5.

The experimental results present also an agreement between the measured profiles and the expected angular response provided by the modeling introduced in this paper. Modeling properly the angular response of the suggested tag is relevant for different aspects. As described in the section “LEO satellite identification: end-to-end performance analysis”, the angular response of the tag influences the extent of the visibility window of each satellite, and therefore the amount of available attempts to make an interrogation. Moreover, non-operational satellites or debris objects are likely to spin while orbiting around the Earth. As a consequence, the return signal will have a time modulation determined by the combination of the spin movement and of the tag angular response (an example of this effect is described in Supplementary Note 3). It is important to have a proper knowledge of this modulation, to be able to compensate for measurement artefacts, especially in case of tightly-packed spectral channels. On the other hand, the presence of this characteristic modulation effect could enable additional information retrieval methods, since the time evolution of the return signal is related to the spinning characteristics of the satellite.

Additionally to the cooperative identification, the selected tag architecture of SLP is open for the implementation of other services from ground, for example satellite laser ranging and satellite attitude characterization. These features have not been investigated in detail in this study, but they represent natural development avenues of the concept, since the SLP method is enabled by retroreflector technology and radiometric detection. Especially for the satellite attitude characterization, one option could be to mount a different number of tags on the different faces of the satellites, similar to what is proposed in^7^, and by observing the expected variations of the measured signals over multiple passes, retrieve information about the satellite attitude.

In conclusion, the method proposed is a viable solution for a cooperative identification of satellites employing optical technologies. This cooperative identification can be implemented as an additional service on existing OGS’s, complementing the already available services like SLR^16^, optical communication^17^, or optical satellite observations^18^. The initial end-to-end simulations described in this paper show that a feasible reconstruction is possible using state-of-the-art laser power levels and detectors. Similar type of components are already integrated in systems employed for the previously mentioned applications. The high degree of compatibility between the different applications, and the possibility to introduce multi-service stations, support the research and the introduction of satellite identification methods, as the SLP concept here described does. The operational outlook of the SLP concept could include the identification of non-responsive/non-operational cooperative satellites; this would allow identifying debris originating from cooperative satellites. Furthermore, it also enables monitoring swarm satellite deployment, which is currently difficult to predict or monitor from ground^19^.

## Methods

### End-to-end simulations

The numerical model built to run the simulations described in the section “LEO satellite identification: end-to-end performance analysis” wisely arranges several analytical models available in literature. The trajectory of a circular LEO satellite is calculated employing analytical models of orbit propagation reported in^20^. The satellite is assumed to have an altitude of 500 km and an inclination of 82.907^∘^. To calculate the viewing geometry from ground, it is assumed that the OGS is situated at (52.1098^∘^ N, 4.3275^∘^ E). The simulations consider a given time date and calculate the evolution of the satellite on its orbit, and the viewing geometry with respect to the OGS. At this stage the orbital evolution is coarsely sampled to locate a time window in which the satellite is visible and a detectable signal can be returned from the tag. The satellite body is assumed to consist of 4 lateral faces and to be constantly pointing in Nadir direction. Based on this assumption, the AOI on each of the satellite faces is calculated, and a visibility time window is defined as the time interval in which at least one face is visible from the OGS, with an angle of incidence on the OGS smaller than 20^∘^. This angle of incidence corresponds to a reduction of about −6 dB in the optical cross-section of a single CCR^15^. For the case reported in Fig. 3 the visibility window extends to about 45 s.

The second step of the simulation is a time-event simulation run over only the visibility window. The event characterizing the simulation is a single pulse of the pulsed lasers used to generate the signal. The pulsed lasers have pulse energies of 100 μJ per spectral channel, pulse durations of 10 ns, and a repetition rate of 10 Hz, with spectral channels uniformly distributed over the bandwidth 1535–1580 nm. The laser parameters are typical values of commercially available pulsed lasers in C-band, except for the repetition period, which could typically reach kHz range. However, in this case the repetition rate was limited to 10 Hz to limit the computation time. The detailed model of SLR end-to-end propagation^15^ is used in this work. The CCR array Far Field Diffraction Pattern is calculated summing up incoherently the patterns of a single retroreflector. This assumption is valid in the case of large number of simulated pulses, as suggested in ref. ^15^. At this stage of the simulation, only the angular response due to the geometry of the CCR is considered. The additional signal damping described in Equation (5) is not considered in the simulation.

The detection chains of the SLP OGS system are modeled independently for each spectral channel. The detection model considers an Avalanche Photo-Detector (APD), a Capacitive-Transimpedance Amplifier (CTIA) and a block for Analog-to-Digital Conversion (ADC). The opto-electronic and noise models for the APD and CTIA are based on ref. ^21^ and ref. ^22^. The choice for the CTIA is made upon considerations of dark noises present in the typical detectors and the expected strength of the return signals. On top of electronic noise contributors, background light sources are considered. To model the background radiation impinging on the OGS telescope, the spectral radiance data from the spectra recorded at Lake Montauban, Canada, case 1, from ref. ^23^ are considered. Therefore, the cases represented by the simulations are limited to night time with limited cloud coverage.

Once the digitized spectral signals are calculated in the model they are averaged. Finally the spectral signature for each configuration simulated is calculated. In the numerical model the spectral signature is calculated as just as a simple ratio between the measured signal intensity at different spectral channels. The spectral signature as formulated in Equation (3) represents an improvement in terms of error propagation, however, at the time of the modeling development this feature has not yet been included. Nevertheless, it is expected that by using the formulation of (3) results could be improved with respect to what reported in 2.2. According to combinatorial theory, the dimension of the spectral signature vector is given by the number of unique ratios between the spectral channel, which is given by

8 $$di{m}_{\rho }=\left(\begin{array}{c}{L}_{\lambda }\\ 2\end{array}\right)$$

For the configuration under test, the spectral signature is a vector in a 6-dimensions space. For the application of the ML approach, for each calculated received spectral signature, the distance of this vector in a 6D space with respect to the all 35 nominal signatures is taken. The nominal dictionary signatures have been calculated assuming ideal BPF responses with in-band transmission and negligible cross-coupling. Therefore the components of the nominal signatures are calculated as

9 $${\rho }_{i| j}=\frac{{S}_{i}}{{S}_{j}}\approx \frac{{N}_{i}+1}{{N}_{j}+1}\quad i=1,\ldots ,{L}_{\lambda }-1,j=i,\ldots ,{L}_{\lambda }$$

The assumption of ideal BPF response is not far from reality since custom filter design could be employed for this application, easily reaching values of *T*^2^ = 0.98^2^ ≈ 1 and ${C}^{2} < {({1}^{-3})}^{2}\, \approx \, 0$.

The values of the stochastic variables have been extracted from normally distributed random realizations, characterized by specific standard deviations which are provided as input parameters of the simulation (see Supplementary Note 1 and SI-Table 1 for a full overview of the input parameters used).

### Ground-to-ground free space test: system description

**Fig. 8:**
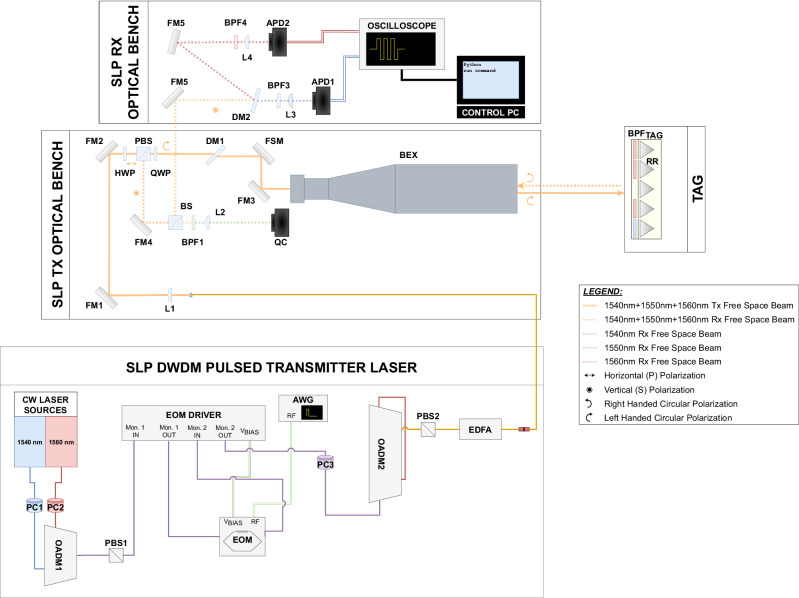
Block diagram of the ground-to-ground free space test. The diagram, not in scale, shows the three main subsystems of the SLP transceiver (Pulsed Transmitter Laser based on fiber components), the Tx Optical Bench and the Rx Optical Bench, both based on free space optical components.

The complete block diagram of the ground-to-ground test setup is shown in Fig. 8. The first subsystem is represented by the Dense Wavelength Division Multiplexing (DWDM) pulsed laser transmitter. The continuous wave (CW) outputs of two Distributed Feedback Bragg lasers, *Thorlabs WDM8-C-12C-20-NM, WDM8-C-37C-20-NM* are multiplexed with an Optical Add-Drop Multiplexer, *FS 169159*. The DWDM multiplexed signal is then directed towards a modulation stage. This is implemented with an electro-optical modulator (EOM), *Thorlabs LNLVL-IM-Z*. The bias voltage is provided to the EOM by a bias driver module, *Thorlabs MBX*. The bias driver implements a feedback loop to have a stable operation point for the EOM. The modulation waveform is provided to the EOM with an Arbitrary Waveform Generator (AWG), *Tabor P9484D*. Although not shown in Fig. 8 a radiofrequency (RF) amplifier, *MiniCircuits ZHL-6A-S+*, is presented to condition the modulating signal voltage before entering the EOM. The output of the EOM is then directed towards the last amplification stage. However, as shown in Fig. 8, before reaching the Erbium-Doped Fiber Amplifier (EDFA), the signal passes again through a second OADM, *FS 169159*. This second OADM is present to provide the possibility to multiplex a third CW channel, for calibration purposes. This additional channel was not used during the ground-to-ground tests, and therefore not shown here. On the signal generation chain the second OADM does not introduce any modification to the signal, since the pulsed spectral channels are first dropped, and then added on the main line. The losses introduced by this extra passage are compensated by the final EDFA stage, *Amonics AEDFA-PM-UL-33-R-FA*. In between the components of the transmitter subsystem, several in-fiber polarization controllers (PC), *Thorlabs CPC900*, are used to align the polarization state of the light in those components which have non-polarization maintaining fibers.

The amplified multiplexed signal is then delivered to the optical bench of the SLP OGS transceiver. The light exiting the fiber is collimated by an aspheric lens (L1), *Thorlabs AL2550J-C, f = 50 mm*. The collimated beams are routed by means of two folding mirrors (FM1,FM2), *Thorlabs PF20-03-P01*. A half-wave plate (HWP), *Thorlabs WPH10M-1550* is used to align the polarization state of the beams with respect to the crystal axes of a polarization beam splitter (PBS), *Thorlabs PBS254*, then a quarter-waveplate (QWP), *Thorlabs WPQ10M-1550* transforms the polarization state of the beams in circular left-handed. After the QWP, the beams are transmitted by a dichroic mirror (DM1), *Thorlabs DMLP950L*, and routed by a fast steering mirror (FSM), *Optics In Motion OIM102* and an additional folding mirror (FM3), *Thorlabs PF20-03-P01*. An afocal beam expander (BEX), *Special Optics 50-130-10XAP*, provides a magnification of ×10. For the ground-to-ground test the optical powered components, L1 and BEX, have been aligned to launch a slightly divergent beam, characterized by a half-angle 1/*e*^2^ divergence of 70 μrad and a 1/*e*^2^ beam radius at the aperture of 45 mm, these two values have been optimized considering the expected pointing stability of the full assembly.

The tag under investigation is composed by an array of 5 CCRs, *Thorlabs PS975-M01B*. To implement the different dictionary words, two classes of BPFs are used, *Thorlabs FBH1540-12* for 1540 nm, and *Thorlabs FBH1560-12* for 1560 nm.

The receiving subsystem of the SLP OGS system interfaces with the transmitter at the PBS. The returning light is reflected by the PBS, routed through two folding mirrors (FM4,FM5), and a 30–70% beam splitter (BS), *Thorlabs BS081*. A bandpass filter, mounted with an angle of incidence of about 20^∘^ is used as dichroic mirror (DM2), *Semrock FF01-1538/82-25*. The angle of incidence is aligned to optimize transmission and reflection, respectively, of the two spectral channels^12^. The spectrally separated beams are then directed towards two identical branches composed by bandpass filters (BPF3, BPF4), *Thorlabs FBH1540-12* and *Thorlabs FBH1560-12*, focusing lenses (L3,L4), *Thorlabs LA1131-C-ML*, and detectors (APD1,APD2), *Thorlabs APD450C*. An oscilloscope, *Rigol DS4034*, is used for the acquisition of the voltage signals. The oscilloscope acquisition is controlled by means of an in-house developed Python script.

In Fig. 8, additional free space optical ports are shown, namely the reflecting port of the DM1 and the transmissive port of the BS. These ports are used in other transceiver configurations which are independent from the SLP test. The FSM in the transceiver is connected to the quad-cell (QC) present in the bench, to provide a feedback loop for tracking. During the SLP tests the FSM was only used in open loop to reach fine alignment with the target.

### Ground-to-ground tests: procedures

The initial alignment at the test location involves aligning the OGS transceiver beam to the tag target location. This is done in two steps: a coarse visual alignment, where the OGS support mount was manually adjusted to have the OGS BEX line of sight oriented towards a target flashlight placed at the target site. For the fine alignment, the target consists of a large retroreflector (aperture diameter 70 mm). Using a single laser beam from the transmitter, the BEX beam is angularly scanned by means of a programmed FSM movement, until the maximum back-reflected power is found on the transceiver detectors.

Once the transmitted light is correctly received, the modulation of the multiplexed light is turned on. The light is modulated by a 5 MHz square wave (50% duty cycle) for 16 μs in round A and 8 μs in round B, followed by 32 μs of no modulation. An example of one waveform is reported in SI-Fig. 3 of the Supplementary Note 4.

These specific times are chosen such that the reflected signal shows three distinct regions. When the 5 MHz clock is sent, a back-reflection from the BEX and other internal components of the transmitter path occurs. This is measured in different proportions on both APD’s, depending on the frequency and the internal alignment. However, most of the light is transmitted and subsequently reflected by the tag at 2.45 km. Only background noise is measured by the APD’s during a time period of about 16.3 μs, corresponding to the time of flight of the burst signal. After the time of flight interval, the retroreflected pulse arrives back at the transmitter and it is measured on both APD’s. The outputs of the APD’s are connected to two of the four channels oscilloscope, which internally averages the signals 2048 times. These averaged waveforms are captured and saved locally to be later used for further analyses. For the plots reported in Fig. 5 a total of 20 waveforms were collected.

A dictionary run starts by first having no filters in placed at the tag. Then, from left to right (always skipping the retroreflector in the center), 1540 nm (1) and 1560 nm (2) filters are placed in front of the retroreflectors in the tag in different configurations with the following order: [ × × × × × ] ({00}), [1 1 × 1 1] ({40}), [1 1 × 1 2] ({31}), [1 1 × 2 2] ({22}), [1 2 × 2 2] ({13}), [2 2 × 2 2] ({04}), where the symbol × indicates a retroreflector without BPF.

Regarding the angular response, the same waveform scheme is used to modulate the laser light. The tag was mounted on a rotational mount and rotated in the azimuthal plane with steps of 2^∘^. In between two angular steps, with an interval of about 5 s, a single averaged waveform was acquired.

### Ground-to-ground tests: data analysis

A full dictionary run consists of acquisition of an equal number of waveforms per wavelength. All the 5 tag configurations are tested with the full dictionary run The algorithm for each waveform is the same for both spectral channels: first the two time intervals relative to the back-reflected pulse and the retroreflected pulse are extracted from the waveform. Next, a third-order polynomial (*a*_0_ + *a*_1_*x* + *a*_2_*x*^2^ + *a*_3_*x*^3^) is fitted to these signals and then subtracted from them. This step allows to remove slow time drift due to the bandwidth limitations of the combined transceiver. The root-mean-square (RMS) is calculated from the remaining signal. Therefore, for each configuration, two sets of data are available: ${B}_{i,j}^{nm}$, the RMS value of the backreflected pulse for the configuration [*nm*], and the corresponding signal reflected from the tag, ${S}_{i,j}^{nm}$. The indices {*i*, *j*} denote, respectively, the number of waveform in the acquired dataset (*i* = 1, …, 20 for Round A, = 1, …, 100 for Round B), and the spectral channel (*j* = 1 for 1540 nm channel and *j* = 2 for 1560 nm channel). The nominal signature for the tested SLP system should be calculated according to Equation (3). However, during the measurements, a dynamical variation of the relative return signals is observed. This variation is ascribed to time-dependent fluctuation of the free space channels, and drift in the OGS DWDM Pulse Transmitters (polarization drifts and gain drifts in the amplifying stage). In order to compensate for these dynamic changes the signature is obtained employing normalized versions of the return signals. This normalized quantity is expressed as

10 $${r}_{i,j}^{nm}=\frac{{S}_{i,j}^{nm}}{{B}_{i,j}^{nm}}.$$

In addition, when initially tested with the configuration {00}, an unbalance between the received return signals is observed. This unbalance is ascribed to the non-uniform gain curve of the EDFA. To compensate for this unbalance, the calibration factor *κ* is added. After these two compensation procedures, the final signature can be calculated as follows,

11 $${\rho }_{i,1| 2}^{nm}=\frac{{r}_{i,1}^{nm}-\kappa {r}_{i,2}^{nm}}{{r}_{i,1}^{nm}+\kappa {r}_{i,2}^{nm}}$$

To determine *κ*, the data from the {00} tag-configurations is used, since tag and channel conditions should be the same for both wavelengths. The ratios between the measured signal at 1540 nm and 1560 nm are used to compensate for the relative difference between the two channels,

12 $$\kappa =\left\langle \frac{{r}_{1}^{00}}{{r}_{2}^{00}}\right\rangle =\frac{1}{N}\mathop{\sum }\limits_{i=1}^{N}\frac{{S}_{i,1}^{00}}{{S}_{i,2}^{00}}\frac{{B}_{i,2}^{00}}{{B}_{i,1}^{00}}$$

It must be highlighted that the compensation procedure applied for the experimental data became necessary after the system was already installed. In future design, this compensation procedure should be replaced by an in-line calibration optical branch, which should provide a live diagnostics on the interrogation signals. The procedure here described is functionally equivalent to an in-line calibration, as it attempts to compensate static and dynamic unbalances on the spectral channels, by exploiting the available information on the internal backreflection and reference targets (configuration {00}). The confusion matrices of Fig. 6 are built for each tag configuration by reporting the frequency of a predicted identifier as function of the actual tested identifier^24^.

### Supplementary information

Supplementary Information

## Data Availability

The datasets generated during and/or analyzed during the current study are available from the corresponding author on reasonable request.

## Code availability

The Matlab and Python scripts used for the current study are available from the corresponding author on reasonable request.
