# Supplementary material for: Satellite License Plate: passive and compact optical spectrally-based identification method for satellites
Source: Commun Eng. 2024 Mar 20;3:41. doi: 10.1038/s44172-024-00188-2 (PMC10955871; doi:10.1038/s44172-024-00188-2)
Supplement: Supplementary file 1 — Supplementary Information [file 44172_2024_188_MOESM1_ESM.pdf]

SUPPLEMENTARY INFORMATION

Satellite License Plate: passive and compact optical spectrally-based identification method for satellites

1 Supplementary Note 1: End-to-end performance analysis - Model Input Parameters

The results shown in section 2.2 of the paper have been obtained with the configuration of input parameters reported in SI-Table 1.

2 Supplementary Note 2: Tag Angular Response

The angular response of the tag is characterized by two combined effects, the reduction of the effective aperture of the CCR, which reduces the effective optical cross section of the tag [1], and the angular response of the BPF. Both effects will determine a reduction of the available retroreflected light as the angle of incidence is increased. For the first effect, the modelling framework explained in [1] is used both to simulate the end-to-end performance of the LEO satellite case, section 2.2 of the paper, and to compare the experimental results to the theoretical geometric reduction in the signals, see section 2.4 of the paper. The experimental results show a smoother peak in the neighborhood of normal incidence, than what is predicted by only geometric arguments. The smooth profile is determined by the coating angular response of the BPF. To model this, it is assumed that the spectral response of a BPF could be approximated with a Gaussian behaviour

$$T(\lambda) = \exp\left[-2\frac{(\lambda-\lambda_o)^2}{\mathrm{BW}^2}\right] \tag{1}$$

with λ_o central wavelength of the BPF, and BW the $1/e^2$ half-bandwidth. When a multilayer coating is illuminated at an angle different from normal incidence, in first order, a blue-shift of the transfer function it is expected. This blue-shift can be modelled with the introduction of the effective refractive index n_{eff}, as introduced in [2]

$$\lambda_o(\theta_{inc}) = \lambda_o\sqrt{1-\left(\frac{\sin(\theta_{inc})}{n_{eff}}\right)^2} \approx \lambda_o\left[1-\frac{1}{2}\left(\frac{\sin(\theta_{inc})}{n_{eff}}\right)^2\right] \tag{2}$$

When Equations SI-1,2 are combined, the same profile as suggested in section 2.2 is obtained. With the approximation that $\frac{\sin(\theta_{inc})}{n_{eff}} << 1$, which is true with good approximation for the considered case, $\theta_{inc} < 30°$ and $n_{eff} = 2.5$, the following is true

$$\lambda - \lambda_o \approx \lambda - \lambda_o \left[1 - \left(\frac{\sin \theta_{inc}}{n_{eff}}\right)\right] \quad (3)$$

The effect of the blue-shift on a single spectral channel is evaluated, considering the transmission coefficient at the central wavelength λ_o as function of the incident angle θ_{inc}. With the approximation of Equation SI-3 this becomes

$$T(\lambda_o; \theta_{inc}) = \exp\left[-\frac{\lambda_o^2 \left(\frac{\sin \theta_{inc}}{n_{eff}}\right)^4}{2\mathrm{BW}^2}\right] \quad (4)$$

3 Supplementary Note 3: Effect of Tag Angular Response in presence of Satellite Spinning

The angular response of the tag has an impact on the detection. The return signal will experience a modulation due to the slant range and due to varying angle of incidence of the illumination beam over the tag as the satellite moves along its orbit (both profiles are visible in Fig. 3 of the of the main text). The same effect applies in the case in which the satellite is spinning: this induced modulation on one side reduces the average signal return strength over the visibility time window (or, equivalently, reduces the time interval of observation, and the available data collected on a single path), but on the other hand it also carries relevant information about the spinning characteristics of the spacecraft. To visualize these two consequences, a simple model is established, where one assumes that the satellite spins around its major axis of inertia with a given spinning rate. According to the simulation framework described in section 4.1 of the main text, a satellite with 4 tags mounted on the 4 lateral faces has its major axis of inertia parallel to the local tangent orbit trajectory. On top of its natural orbital evolution, one assumes that the satellite spins around its axis with a rotational speed of $6\,(°/s)$; this rate is in agreement with observations reported in [3], where the rotation period of CZ-3B R/B is observed to be confined between $[60, 70]\,(s)$. According to the initial simulations performed in Section 2.2 of the main text, without any spin movement, the satellite is visible for an interval of about $45\,(s)$. Over this interval, due to the assumed spin, the satellite exposes 3 faces to the illumination beam. The effective return signal, modulated by the angular response, will be effectively similar to what is reported in SI-Fig. 1, where, for the evolution of the signal trace, the formulation proposed in Equation SI-4 is used, combined with the geometrical modelling proposed in [1]. The graph depicted in SI-Fig. 1 clearly shows that there is a reduction of the signal strength due to the spinning of the satellite and an average effective return of 20% of the maximum signal is expected. In case the detection sensitivity threshold is set around 20%, the effective time in which a satellite is visible reduces to about $< 14\,(s)$, which is not surprising, since the acceptance cone (defined as the angular range for which the normalized return is $> 20\%$) is

about 27°, corresponding to one-third of the full angular sector for a single face (90°). From a system design point of view, if this additional penalty cannot be absorbed at system level, for example by increasing the available output power at the transmitter, it will lead to a reduction of the available interrogation time. This can nevertheless be compensated by multiple observation over several passes, since it allows for the collection of larger amounts of data, increasing the yield of the decoding approach.

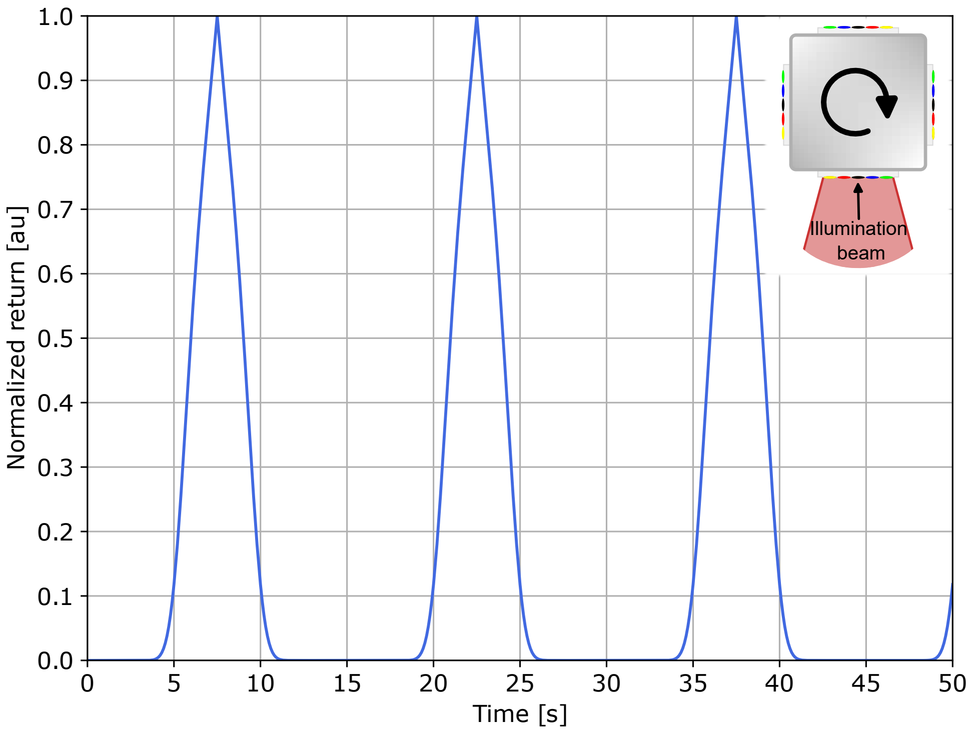

SI-Fig. 1 Time evolution of the normalized return signal as effect of the tag angular response combined with a satellite spinning rate of 6 $(°/s)$ around its major inertia axis. In the inset, a sketch of the considered configuration with the cross-section of the satellite spinning around its axis and a tag on each of the lateral faces.

It should be noted that, although posing yet another challenge for the link budget of the proposed method, the angular response of the tag can also be exploited to gather additional information on the satellite characteristics. A time trace of a single pass will be characterized by the presence of characteristic harmonics related to the spinning period and the number of faces on which tags are mounted. Even in presence of noisy traces, this behaviour can be extracted, and information about the spinning rate can be extracted. As a qualitative example, a modulated time trace with superimposed noise is shown in SI-Fig. 2, along with its frequency spectrum. The additive simulated noise is characterized by a large amplitude compared to the signal scale,

with a standard deviation $\sigma = 27\%$, meaning that, in the worst statistical case, a noise sample can corrupt the received signal by up to 80% of the full scale. Even in this quite extreme case, the modulation induced by the spinning of the satellite is visible in the time domain, and even more clear in the frequency domain, where the harmonics related with the expected period induced by the spinning $(15\,(s))$ are visible. This suggests that, by performing Fourier analysis on the received traces, the spinning rate of the satellite under investigation can be inferred. This is definitely an important aspect as it increases the amount of information that can be extracted by the SLP method.

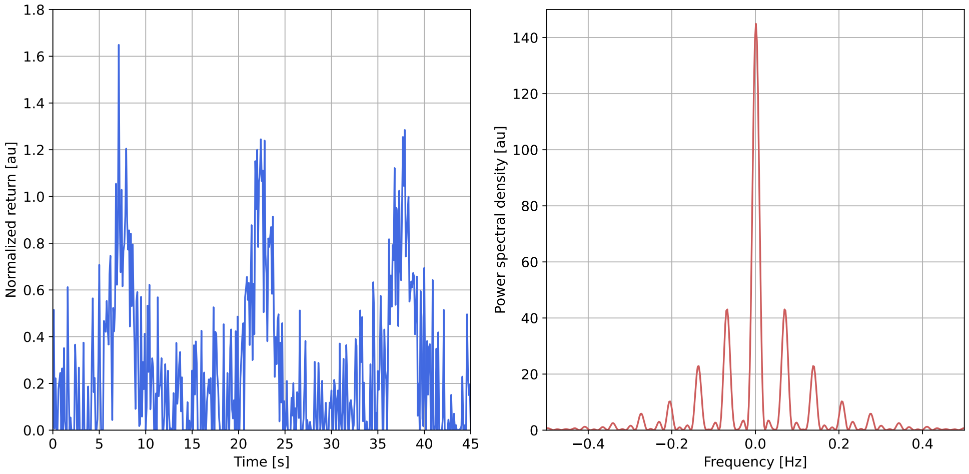

SI-Fig. 2 Time evolution of the return signal, as in SI-Fig. 1, corrupted by a white noise with Normal distribution and standard deviation normalized to $\sigma = 27\%$ of the full scale.

4 Supplementary Note 4: Experimental waveform data

An example of an experimental waveform as recorded during the test is shown in SI-Fig. 3. The segmentation process described in section 4.4 of the paper was applied on this type of waveform to separate the backreflection from the return signal contribution. Moreover, the same waveform were used during the test campaign for a time-of-flight type of experiment to assess the actual distance between the two test sites. It can be seen in fact that the initial wavefront of the two burst is separated by $16.25\,\mu$s, which corresponds to a distance of 2.43 km.

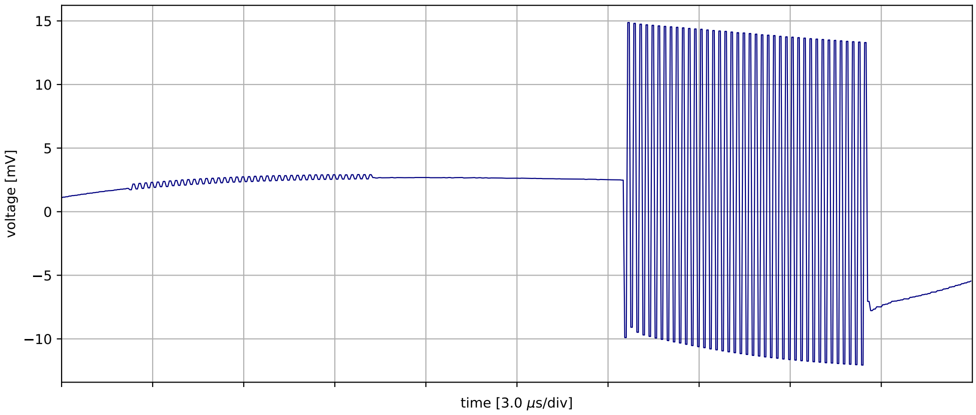

SI-Fig. 3 Example of single channel waveform recorded during the ground-to-ground free space test. The backreflection burst (first from left) and the return signal burst (second from left) are clearly visible in the waveform.

5 Supplementary Note 5: Statistical analysis of experimental data

Starting from the Confusion Matrices developed for the statistical analysis of the experimental data, several measures could be derived. Amongst these in Table 2 the values of Sensitivity, Precision, and Matthew's Correlation Coefficient (MCC) according to the formalism developed in [4], are reported. The first two parameters quantify, respectively, the positive correctly classified samples to the total number of positive samples, and the proportion of positive samples that were correctly classified to the total number of positive predicted samples. The third parameter, the MCC, reports the predictor's performance.

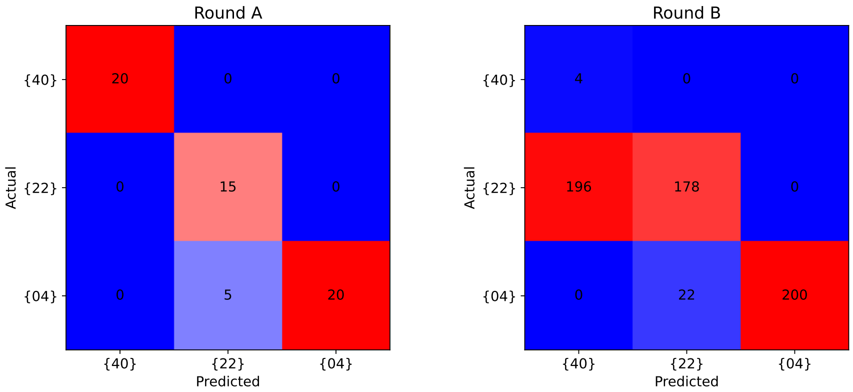

SI-Fig. 4 The confusion matrix for both rounds A and B, in the case of a reduced dictionary employing only [{40}, {22}, {04}]. The x-axis shows the predicted identifier tag and the y-axis the actual identifier tag.

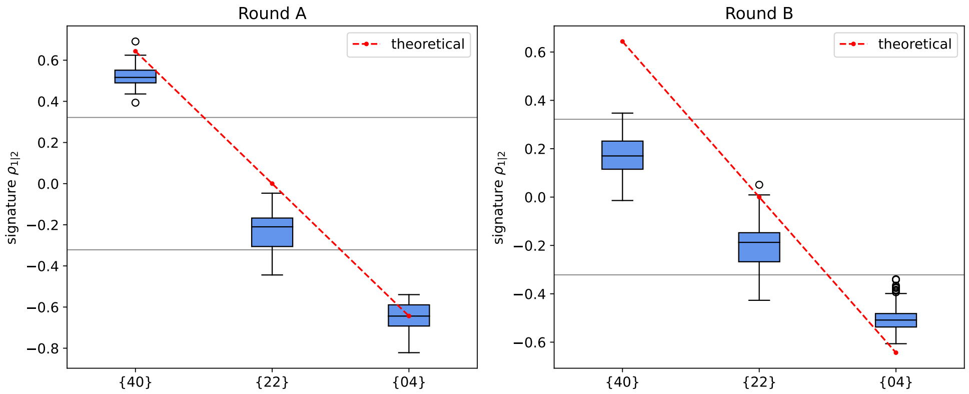

SI-Fig. 5 SLP Dictionary Test results, as reported in Fig. 6 in the article, but this time for the reduced dictionary case employing only [{40}, {22}, {04}]

The same type of metrics are reported in Table 2 for the case where a reduced dictionary, composed from only three identifiers, is used. For the same case also the Confusion Matrices, SI-Fig. 4, and the dictionary boxplots, SI-Fig. 5, are reported. In this reduced case the metrics are improved, showing that in principle the method is valid for identification, although in case of high system uncertainty, like in round B, small granularity dictionaries are not possible.

Supplementary References

[1] Degnan, J. J. *Millimeter Accuracy Satellite Laser Ranging: a Review*, 133–162 (American Geophysical Union (AGU), 1993). URL https://agupubs.onlinelibrary.wiley.com/doi/abs/10.1029/GD025p0133. https://agupubs.onlinelibrary.wiley.com/doi/pdf/10.1029/GD025p0133.

[2] IDEX, H. . S. L. Filter spectra at non-normal angles of incidence URL https://www.idex-hs.com/resources/resources-detail/filter-spectra-at-non-normal-angles-of-incidence.

[3] A.Vananti, M. R. P. D.-M. J., D.Kucharski *Tumbling motion assessment for space debris object* (2022). URL https://nebula.esa.int/sites/default/files/neb_tec_studies/2745/public/GT17-152GR_EX.pdf.

[4] Tharwat, A. Classification assessment methods. *Applied Computing and Informatics* **17**, 168–192 (2021). URL https://doi.org/10.1016/j.aci.2018.08.003.

SI-Table 1 Input data used for running the simulations of section 2.2

Channel

Parameter	Unit	Value
Two-way Atmospheric Transmission	(dB)	-0.4
Fried Parameter	(mm)	166
Background Spectral Radiance	(W m^{-2} sr^{-1} nm^{-1})	9e-08
Mean Cirrus Cloud thickness	(m)	1341

OGS

Parameter	Unit	Value
OGS Longitude	(°)	4.3275
OGS Latitude	(°)	52.11
OGS Max Elevation	(°)	85
OGS Min Elevation	(°)	5
OGS Height on Sea Level	(m)	65

Satellite

Parameter	Unit	Value
Satellite Altitude	(km)	500
Satellite Longitude Ascending Node	(°)	100
Satellite Inclination	(°)	82.907

SLP Tag

Parameter	Unit	Value
Retroreflector Aperture Radius	(mm)	6.35
Retroreflector Double-Pass Wavefront Error	(nm)	24
Retroreflector Refractive Index	(-)	1.45
Retroreflector Reflection	(-)	0.99
Bandpass In-Band Transmission	(-)	0.9
Bandpass Out-Band Max Transmission	(-)	0.001
Tag Available Spectral Channels	(-)	4
Tag Available Relative Return Levels	(-)	4
Tag repetitions over the satellite body	(-)	4

OGS Telescope

Parameter	Unit	Value
Rx Telescope Diameter	(mm)	800
Rx Telescope Obscuration	(%)	40
Rx effective focal length	(mm)	2000
Rx On-Sky Field of View - Half-Angle	(μrad)	50
Rx f-number	(-)	2.5
Rx Optical Transmission Losses	(dB)	-2.1
Rx Filter FWHM Passband	(nm)	3

OGS Transmitter

Parameter	Unit	Value
Wavelength	(nm)	1535-1580
Laser Pulse width	(ns)	10
Laser Pulse Energy	(mJ)	0.1
Laser Repetition Rate	(kHZ)	0.01
Beam Half-Angle Divergence	(μrad)	70
Optical Transmission loss	(dB)	-2
Beam Wavefront Error	(nm)	200
Static pointing error 1-sigma	(μrad)	20
Dynamic mechanical pointing error 1-sigma	(μrad)	10

OGS Detection Chain

Parameter	Unit	Value
APD Responsivity	(A W^{-1})	0.8
APD Expected M Gain Factor	(-)	30
APD Excess Noise Factor Index	(-)	0.7
APD Dark Current	(nA)	40
APD Internal Capacitance	(pF)	10
APD Shunt Resistance	(MΩ)	10
CTIA Input Spectral Voltage Noise	(nV $Hz^{-1/2}$)	0
CTIA Feedback Capacitance	(pF)	10
CTIA Input Bias Current	(pA)	0
CTIA Operation Temperature	(K)	300
Detection Gating Time	(μs)	1
ADC Input Saturation Voltage Level	(V)	2.5
ADC Input Amplifier Gain	(-)	200
ADC Bit Resolution	(bit)	24

Round A - Full Dictionary			
Tag configuration	Sensitivity	Precision	Matthew's correlation coefficient
{40}	80%	57.1%	0.5790
{31}	40%	66.7%	0.4308
{22}	25%	71.4%	0.3527
{13}	75%	50.0%	0.4910
{04}	100%	87.0%	0.9149
**Average**	**64%**	**66.4%**	**0.5537**

Round B - Full Dictionary			
Tag configuration	Sensitivity	Precision	Matthew's correlation coefficient
{40}	0.0%	0.0%	0.0000
{31}	29.5%	34.3%	0.1630
{22}	31.5%	21.6%	0.0264
{13}	82%	46.5%	0.4886
{04}	74%	80.4%	0.7174
**Average**	**43.4%**	**36.6%**	**0.2791**

Round A - Reduced Dictionary			
Tag configuration	Sensitivity	Precision	Matthew's correlation coefficient
{40}	100%	100%	1.0000
{22}	75%	100%	0.8165
{04}	100%	80%	0.8367
**Average**	**91.7%**	**93.3%**	**0.8844**

Round B - Reduced Dictionary			
Tag configuration	Sensitivity	Precision	Matthew's correlation coefficient
{40}	2%	100%	0.1159
{22}	89%	47.6%	0.3891
{04}	100%	90.1 %	0.9227
**Average**	**63.7%**	**79.2%**	**0.4759**

SI-Table 2 Statistical metrics derived from the Confusion Matrices, for the two test rounds, when the full or reduced dictionaries are used for the identification.
